# Endophytic microorganisms as sustainable sources of antidiabetic metabolites: biochemical mechanisms, bioprospecting strategies, and translational challenges

**DOI:** 10.1007/s13205-026-04928-3

**Published:** 2026-06-25

**Authors:** Sherif B. Adeyemi, Mercy A. Alabi, Hafizah Y. Chenia, Tricia Lin, Johnson Lin

**Affiliations:** 1 Discipline: Microbiology, School of Life Science, College of Agriculture and Science, University of KwaZulu-Natal, Private Bag X54001, Durban, 4000 South Africa; 2 Department of Microbiology, Stellenbosch University, Private Bag X1, Matieland, 7600 South Africa; 3 Tallaght University Hospital, Tallaght, Dublin 24, D24 NR0A Ireland; 4 Department of Plant Biology, Faculty of Life Sciences, University of Ilorin, PMB 1515, Ilorin, Kwara State Nigeria

**Keywords:** Diabetes mellitus, Type 2 diabetes, Endophytes, Antidiabetic metabolites, Bioprospecting, Multi-target pharmacology

## Abstract

Diabetes mellitus affects over 589 million adults globally, with projections exceeding 900 million by 2050. Conventional antidiabetic therapies are limited by adverse effects, high cost, and single-target pharmacology. Endophytic microorganisms, fungi and bacteria residing asymptomatically within plant tissues, represent an underexplored yet structurally diverse reservoir of bioactive metabolites developed through co-evolutionary adaptation with their hosts. This scoping review aimed to systematically map the breadth and depth of available evidence on: (1) the antidiabetic biochemical mechanisms of endophyte-derived metabolites; (2) bioprospecting and biotechnological discovery strategies; and (3) key translational barriers to clinical application, to identify research priorities and knowledge gaps. A systematic search was conducted across PubMed/MEDLINE, Scopus, Web of Science, and ScienceDirect covering January 2016 to March 2026, supplemented by hand-searching of reference lists. The Boolean search strategy combined endophyte-specific, antidiabetic, and metabolite-related terms. Studies were screened against explicit inclusion and exclusion criteria following PRISMA for Scoping Reviews (PRISMA-ScR) guidelines. A total of 747 records were identified; following duplicate removal and two-stage screening, 123 studies were included in the final synthesis. Included studies documented seven principal antidiabetic mechanisms of endophyte-derived metabolites: (1) inhibition of α-glucosidase and α-amylase; (2) enhancement of GLUT4 translocation via the IRS1/PI3K/Akt/GSK3β axis; (3) AMPK activation suppressing hepatic gluconeogenesis; (4) PPARγ agonism improving insulin sensitivity; (5) antioxidant protection of pancreatic β-cells from ROS-induced apoptosis; (6) anti-inflammatory modulation of TNF-α, IL-6, and IL-1β via NF-κB/MAPK pathways; and (7) multi-target engagement. Bioprospecting strategies, including OSMAC, genomic mining, co-culture systems, and AI-assisted discovery, are accelerating compound identification. Key translational barriers include inconsistent metabolite yields, strain instability, regulatory complexity, and unresolved intellectual property disputes. Endophytic microorganisms constitute a compelling and sustainable source of multi-target antidiabetic compounds. This review provides the first PRISMA-ScR compliant synthesis of the field, offering clinicians, natural product researchers, pharmaceutical scientists, and policymakers a rigorous evidence base for prioritising endophyte-derived lead compounds for preclinical and clinical development. Substantial knowledge gaps remain in the in vivo validation, clinical translation, and equitable access frameworks for microbial resources from biodiverse regions.

## Introduction

Diabetes mellitus is a major chronic metabolic disorder characterised by persistent hyperglycaemia resulting from impaired insulin secretion or defective insulin action, or both. It is associated with progressive damage to multiple organ systems and increases the risk of cardiovascular, renal, neurological and other diabetes-related complications (An et al. 2024; Hacker, 2024; Khalifa And Albadawy 2024). The disease occurs primarily as type 1 diabetes mellitus (T1DM), an autoimmune disorder involving pancreatic β-cell destruction, and type 2 diabetes mellitus (T2DM), which is characterised by insulin resistance and progressive β-cell dysfunction, and eventual loss of glycaemic control (Padhi et al. 2020). T2DM accounts for approximately 90–95% of diabetes cases worldwide and is strongly linked to obesity, sedentary lifestyle, chronic inflammation, oxidative stress and metabolic dysregulation (Lu et al. 2024). Recent estimates indicate that more than 589 million adults are living with diabetes globally, underscoring the need for more effective, accessible, and sustainable therapeutic strategies (Genitsaridi et al. 2026).

The pathophysiology of diabetes, particularly T2DM, involves a complex interplay of insulin resistance, pancreatic β-cell dysfunction, chronic low-grade inflammation, oxidative stress, and dysregulated metabolic signalling pathways, including Phosphoinositide 3-kinase / Protein Kinase B (PI3K/Akt), AMP-activated Protein Kinase (AMPK), and Mitogen-Activated Protein Kinase (MAPK) cascades (Dhas et al. 2024). These intersecting mechanisms create a multifactorial disease landscape that is inadequately addressed by conventional single-target pharmacotherapy. Reactive oxygen species (ROS) contribute to β-cell apoptosis, impaired insulin gene expression, and worsening insulin resistance, thereby accelerating disease progression (Röder et al. 2016; Dludla et al. 2023). Although currently available antidiabetic agents, including metformin, sulfonylureas, Dipeptidyl Peptidase IV (DPP IV) Inhibitors, Sodium-Glucose Cotransporter 2 (SGLT2) inhibitors, and α-glucosidase inhibitors, provide clinically meaningful glycaemic control, their use may be limited by adverse effects, gastrointestinal intolerance, hypoglycaemia, and diminished long-term efficacy, and high cost, particularly in low- and middle-income countries (Padhi et al. 2020; Berbudi et al. 2025). These limitations have intensified interest in alternative and complementary therapeutic sources capable of modulating multiple pathological targets simultaneously.

Natural products remain an important foundation for drug discovery and have contributed substantially to the development of antidiabetic therapeutics (Cragg And Pezzuto 2016; Bailey 2017). Examples include metformin, historically derived from the medicinal plant *Galega officinalis*, and berberine, an alkaloid with documented insulin-sensitising and glucose-loweing effects (Bailey 2017). However, direct reliance on plant-derived metabolites is often constrained by low yield, environmental variability, long cultivation periods, extraction costs, and ecological pressures associated with overharvesting (Nasim et al. 2022; Chaachouay And Zidane 2024). These challenges have shifted attention toward microbial sources of natural products that can be cultivated, optimised, and scaled under controlled conditions.

Endophytes are fungi and bacteria that reside asymptomatically within internal plant tissues and form intimate ecological and biochemical relationships with their hosts (Ashraf et al. 2026). Through long-term co-evolution, endophytes can biosynthesise structurally diverse metabolites, some of which are identical or analogous to those host-derived compounds, while others represent novel chemical scaffolds with distinct biological activities (Rutkowska et al. 2023). These metabolites include alkaloids, polyketides, terpenoids, flavonoids, peptides, and phenolic compounds with potent antidiabetic and anticancer effects (Gouda et al. 2016; Singh et al. 2021). Landmark discoveries such as paclitaxel-producing *Taxomyces andreanae* and camptothecin-yielding *Camptotheca acuminata* have demonstrated the capacity of endophytic microorganisms to serve as alternative microbial platforms for valuable bioactive compounds (Stierle et al. 1993). In diabetes research, endophyte-derived metabolites have been associated with inhibition of α-glucosidase and α-amylase, enhancement of insulin sensitivity, modulation of glucose metabolism, and protection of pancreatic β-cells against oxidative stress (Sharma et al. 2021; Yang et al. 2022).

The biotechnological appeal of endophytes lies in their scalability, metabolic plasticity and, and suitability for strain improvement and fermentation-based production. Unlike medicinal plants, which require long growth periods and are affected by seasonal, geographical and ecological pressures, endophytes can be cultivated under controlled laboratory and industrial conditions, allowing a more sustainable route for continuous metabolite production (Toppo et al. 2023).

Advances in genomics, metabolomics, and synthetic biology are also transforming endophyte-based drug discovery by enabling the identification of cryptic biosynthetic gene clusters, the prediction of bioactive scaffolds, the activation of silent metabolic pathways, and the optimisation of metabolite yield (Burragoni And Jeon 2021). Approaches such as CRISPR-Cas genome editing, heterologous pathway expression, metabolic flux modelling, OSMAC strategies, and multi-omics integration strategies are expanding the chemical diversity accessible from endophytic microorganisms and strengthening their relevance in antidiabetic drug discovery (Maghembe et al. 2020; Ivanisevic And Sewduth 2023).

Despite increasing evidence supporting the antidiabetic potential of endophytic microorganisms, the field remains fragmented across studies reporting diverse host plants, microbial taxa, metabolites, experimental models, and mechanisms of action. A consolidated synthesis is, therefore, needed to clarify the major biochemical mechanisms through which endophyte-derived metabolites exert antidiabetic effects, highlight current bioprospecting and biotechnology-driven discovery strategies, and identify barriers that limit translation into clinically useful therapeutics. This review addresses these issues by evaluating endophytic microorganisms as sustainable sources of antidiabetic metabolites, with specific emphasis on their mechanistic activities, discovery platforms, and translational challenges.

## Methodology

This scoping review was conducted in accordance with the PRISMA extension for Scoping Reviews (PRISMA-ScR) (Tricco et al. 2018) and the Joanna Briggs Institute (JBI) methodological framework for scoping reviews (Peters et al. 2020).

### Eligibility criteria

Eligibility criteria were defined a priori using the Population–Concept–Context (PCC) framework:

Population: Endophytic microorganisms (fungi and bacteria) isolated from any plant host.

Concept: Antidiabetic bioactivity, encompassing enzyme inhibition (α-glucosidase, α-amylase, DPP-IV, PTP1B), insulin sensitisation, GLUT4 modulation, AMPK activation, PPARγ agonism, β-cell protection, and anti-inflammatory cytokine modulation.

Context: In vitro enzyme assays, cell-based models, in vivo animal studies, in silico molecular docking, genomics-guided discovery, and biotechnological optimisation studies.

Included in the study were peer-reviewed primary research articles and authoritative reviews in English; studies reporting antidiabetic bioactivity of endophyte-derived compounds with at least one quantitative measure (e.g., IC_50_, in vivo glucose data, and binding affinity). Excluded resources were conference abstracts without full text; studies focusing exclusively on plant extracts without isolation or characterisation of endophyte components; and articles with no retrievable full text (Table 1).

**Table 1:** Inclusion and exclusion criteria applied to identify eligible studies on endophyte-derived antidiabetic metabolites

Criterion	Inclusion	Exclusion
Study type	Peer-reviewed original research articles on the antidiabetic bioactivity of endophyte-derived compounds	Reviews articles, conference abstracts without full text; non-peer-reviewed reports
Organism focus	Studies characterising fungal or bacterial endophytes from any plant host	Studies focusing exclusively on plant extracts without isolating or characterising the endophyte component
Outcome measure	At least one quantitative antidiabetic measure (e.g., IC50, in vivo glucose data, binding affinity, AMPK activation, GLUT4 translocation)	Studies with qualitative reporting only and no extractable quantitative data
Language	English-language full-text articles	Articles published in languages other than English without a validated English translation
Publication date	January 2016 – March 2026 (primary window); landmark pre-2016 studies of foundational significance included selectively)	Articles published before 2016 unless of foundational significance
Full-text availability	Full text retrievable through institutional or open-access repositories	Articles with no retrievable full text

### Search strategy

A systematic search was conducted across four electronic databases: PubMed/MEDLINE, Scopus, Web of Science (Core Collection), and ScienceDirect. The primary search window covered January 2016 to December 2025, with a secondary update in March 2026 to capture early 2026 publications. Landmark pre-2016 studies of foundational significance were included selectively. Boolean search strings were adapted to each database’s syntax (Table 2).

**Table 2:** Database-specific search strings used to identify studies on endophyte-derived antidiabetic metabolites

Database	String	No
Scopus	TITLE-ABS-KEY ( endophyte* OR endophytic AND fung* OR endophytic AND bacter* ) AND TITLE-ABS-KEY ( antidiabetic OR diabetes OR “insulin resistance” OR glucosidase OR amylase OR GLUT4 OR AMPK OR “pancreatic beta-cell” ) AND TITLE-ABS-KEY ( metabolite* OR “secondary metabolite*” OR “bioactive compound*” ) AND ( LIMIT-TO ( DOCTYPE, “ar” ) ) AND PUBYEAR > 2016 AND PUBYEAR < 2026	94
Pubmed	(endophyte*[tiab] OR “endophytic fung*“[tiab] OR “endophytic bacter*“[tiab] OR “endophytic actinomycete*“[tiab]) AND (antidiabetic[tiab] OR “diabetes mellitus“[tiab] OR “insulin resistance“[tiab] OR “alpha-glucosidase“[tiab] OR “alpha-amylase“[tiab] OR hyperglycaemia[tiab] OR GLUT4[tiab] OR AMPK[tiab] OR “PPARgamma“[tiab] OR “pancreatic beta-cell“[tiab]) AND (metabolite*[tiab] OR “secondary metabolite*“[tiab] OR “bioactive compound*“[tiab] OR “natural product*“[tiab] OR biosynthetic[tiab])	88
WebofScience	TS=(endophyte* OR endophytic fungi OR endophytic bacteria) AND TS=(antidiabetic OR diabetes mellitus OR insulin resistance OR glucosidase OR amylase OR GLUT4 OR AMPK) AND TS=(metabolite* OR secondary metabolite* OR bioactive compound*)	198
Sciencedirect	(“endophyte” OR “endophytic fungi” OR “endophytic bacteria”)AND(“antidiabetic” OR “hypoglycemic” OR “type 2 diabetes”)AND(“metabolite” OR “secondary metabolite” OR “bioactive compounds”)	274
Others	Direct search (endophyte-specific, antidiabetic, and metabolite-related terms)	93

### Selection process

**Fig. 1:**
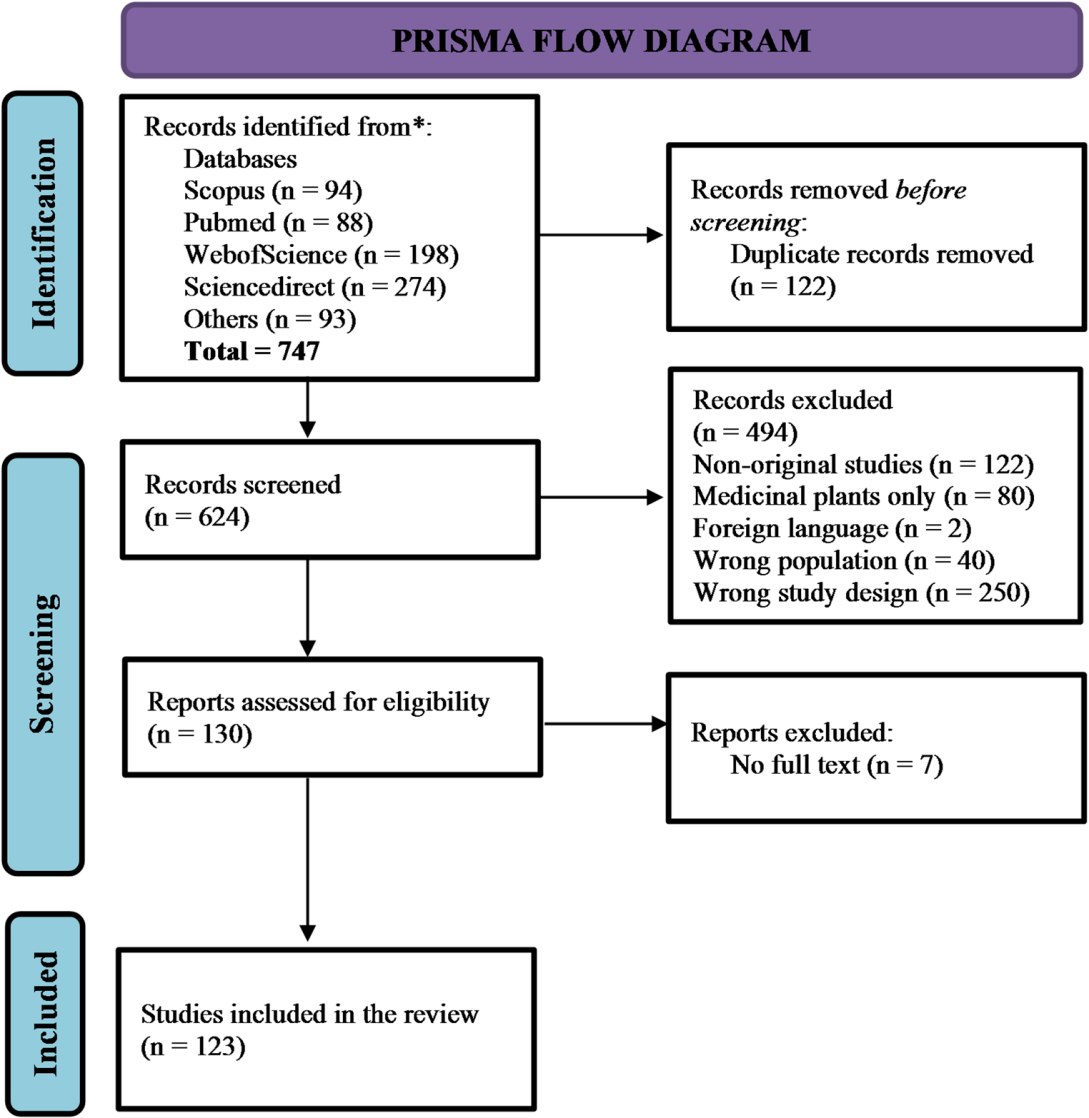
PRISMA flow diagram of the literature selection process. The diagram illustrates the sequential identification, duplicate removal, title and abstract screening, full-text eligibility assessment, and final inclusion of studies. A total of 747 records were identified from electronic database searches and supplementary hand-searching. After removing duplicates, 624 records were screened, and 123 studies met the eligibility criteria and were included in the final review synthesis

Records were imported into the Rayyan systematic review software for duplicate removal and screening. Screening proceeded in two stages: (1) title and abstract screening against the eligibility criteria; (2) full-text review of potentially eligible records. A PRISMA flow diagram documenting the total number of articles retrieved, those excluded at each stage with reasons, and those finally included is presented in Fig. 1.

### Data extraction and charting

Data were extracted by two reviewers using a standardised data charting form piloted on 10 randomly selected articles and refined iteratively. Extracted variables included: first author and year; country of study; plant host; endophyte genus and species; endophyte type (fungal/bacterial); antidiabetic target and mechanism; metabolite class and name where reported; quantitative outcome measure (IC_50_, % inhibition, in vivo glucose data, or binding energy); study design; and evidence quality level. Discrepancies between reviewers were resolved through discussion.

### Evidence synthesis

Given the substantial heterogeneity of study designs, organisms, and outcome measures across the included studies, spanning in vitro enzyme-inhibition assays, cell-based insulin signalling models, in vivo studies, and multi-omics genomic investigations, quantitative pooling (meta-analysis) was neither appropriate nor feasible. Evidence was instead synthesised narratively, organised by antidiabetic mechanism, and supplemented with summary tables. This approach is consistent with JBI scoping review methodology and with established practice for mechanistically heterogeneous fields (Peters et al. 2020).

### Overview of included studies

The search retrieved 654 records from electronic databases and 93 additional records from hand searching, for a total of 747 records. After removing 123 duplicates, 624 records underwent title and abstract screening. Following screening and data extraction, 123 studies met all eligibility criteria and were included in the final scoping review synthesis.

### The endosphere: a source of bioactive secondary metabolites

The endosphere comprises the internal tissues of plants colonised by asymptomatic microorganisms and represents a specialised ecological niche for endophytic fungi and bacteria. Unlike the rhizosphere or phyllosphere, the endosphere offers a relatively stable and protected microenvironment that supports sustained interactions and co-evolution between microbial symbionts and their host plants (Qian et al. 2019). These endophytes reside within roots, stems, leaves, seeds, and reproductive organs without causing disease, and their close association enables extensive biochemical exchange. Through this complex communication, they may modulate host physiological processes and produce structurally diverse secondary metabolites which resemble, complement, or enhance host-derived compounds, a phenomenon known as xenohormesis (Alam et al. 2021; Luo et al. 2025).

The diversity and distribution of endophytic communities are detailed in Table 3, which presents representative examples of fungal and bacterial endophytes, their preferred plant hosts and tissues, and reported functional roles.

**Table 3:** Representative fungal and bacterial endophytes, their plant hosts, tissue distribution, and reported functional roles

Endophyte Group	Dominant Taxa	Preferred Tissue(s)	Common Plant Hosts	Functional Contributions	References
Fungi (Ascomycota)	*Colletotrichum*, *Fusarium*, *Penicillium*, *Aspergillus*	Leaves, stems, reproductive organs	Grasses, tropical trees, cereals	Secondary metabolite production (alkaloids, terpenoids, polyketides), pathogen suppression	Strobel and Daisy (2003); Chauhan et al. (2023)
Fungi (Basidiomycota)	*Cladosporium*, *Cryptococcus*	Leaves, seeds	Woody perennials, cereals	Production of bioactive metabolites, stress tolerance	Roy and Banerjee (2018)
Proteobacteria	*Pseudomonas*, *Burkholderia*, *Enterobacter*	Roots, vascular tissues	Legumes, cereals, horticultural crops	Nitrogen fixation, phytohormone production (IAA, ACC deaminase), disease suppression	Adeleke et al. (2021)
Actinobacteria	*Streptomyces*, *Micromonospora*	Roots, nodules	Legumes, medicinal plants	Antibiotic production, phosphate solubilisation, siderophore secretion	Deltedesco et al. (2020)
Firmicutes	*Bacillus*, *Paenibacillus*	Rhizosphere–root interface, stems	Cereals, oil crops	Induced systemic resistance, biofilm formation, nutrient mobilisation	Chauhan et al. (2023)

### Enzyme inhibitors: α-glucosidase and α-amylase

Inhibition of carbohydrate-hydrolysing enzymes, particularly α-glucosidase and α-amylase, is one of the most extensively documented antidiabetic mechanism of endophyte-derived metabolites in the included literature (*n* > 50 studies). These enzymes mediate the breakdown of complex carbohydrates into absorbable glucose, and their inhibition can reduce postprandial hyperglycaemia (Man et al. 2022). Several fungal and bacterial endophytes have demonstrated inhibitory activity against these targets, supporting their potential as sources of antidiabetic lead compounds.

The endophytic fungus *Chaetomium globosum*, isolated from *Ginkgo biloba* (Qi et al. 2020) produces chaetoglobol acid, a compound with strong α-glucosidase inhibitory activity. Chaetoglobol acid reportedly showed an IC_50_ value of 3.04 µM against α-glucosidase, which is 18-fold higher when compared to acarbose (IC_50_ = 54.74 µM). A moderate inhibitory activity with an IC_50_ of 22.18 µM was also reported against α-amylase (Qi et al. 2020). Similarly, *Aspergillus* and *Penicillium* species isolated from *Momordica charantia* synthesise polyketides and flavonoids with significant α-glucosidase inhibitory activity (Liu et al. 2015). These metabolites also reduce oxidative stress in pancreatic β-cells, mitigating one of the key pathological mechanisms of diabetes (Dludla et al. 2023).

Endophytic fungi associated with *Gymnema sylvestre* further illustrate the enzyme-inhibitory potential of plant-associated microorganisms (Ranjan et al. 2019). Ranjan et al. (2019) isolated 32 fungal endophytes spanning multiple genera and identified *Fusarium equiseti* as a potent producer of mycosterol, a steroidal metabolite with inhibitory activity against α-amylase and α-glucosidase. Mycosterol exhibited IC_50_ values of 4.22 µg/mL for α-amylase and 69.72 µg/mL for α-glucosidase, demonstrating superior α-amylase inhibition relative to the standard acarbose (5.75 µg/mL and 55.29 µg/mL, respectively). Enzyme kinetics studies indicated a competitive mode of inhibition, while molecular docking analyses corroborated strong interactions at catalytic sitesof both enzymes (Ranjan et al. 2019).

Other endophytic fungi have also yielded promising enzyme inhibitors. Nagarajan et al. (2025), using bioassay-directed fractionation of an extract from the endophytic fungus *Penicillium oxalicum* isolated from Cassava root, identified 17 bioactive molecules through GC-MS and LC-MS analysis. Among these, four compounds, viz.; 2-phenylpyrido[3,4-d] − 1,3-oxazin-4-one, guanosine, quercetin-3-O-sophoroside, and esculin demonstrated a strong binding affinity for the PPARγ protein target. *Xylariaceae* sp. QGS 01, isolated from *Quercus gilva* Blume, produced 8-hydroxy-6,7-dimethoxy-3-methylisocoumarine which showed α-glucosidase inhibitory activity (Indrianingsih And Tachibana 2017). Endophytic fungi from *Acacia nilotica* yielded a potent a ~ 22 kDa proteinaceous peptide inhibitor from *Aspergillus awamori* with strong α-amylase and α-glucosidase inhibitory activity (IC_50_ values of 3.75 and 5.625 µg/mL, respectively) and a mixed mode of enzyme inhibition. The inhibitor also demonstrated stability across different pH and temperature conditions, improved activity following process optimisation, and lacked mutagenic effects in Ames testing, highlighting its potential safety as an antidiabetic lead compound (Singh And Kaur 2016). In addition, *Penicillium lanosum* and *Penicillium radiatolobatum* have been reported as sources of multifunctional metabolites, with *P. radiatolobatum* extracts demonstrating α-amylase inhibitory activity (IC_50_ = 362.5 µg/mL), antioxidant capacity (ABTS IC_50_ = 37.5 µg/mL), low cytotoxicity and cytoprotective effects in HEK-293 cells (Naveen et al. 2023). Metabolomic profiling and in silico analyses linked identified compounds, viz.; thiophene A, limonene, and phenylacetic acid, to interactions with key targets involved in diabetes and oxidative stress, reinforcing the therapeutic potential of endophytic fungi as multi-target agents. *Curvularia lunata*, isolated from *Ficus religiosa*, also exhibited marked α-amylase inhibitory activity with an ethyl acetate extract producing 80.40% inhibition and an IC_50_ value comparable to acarbose (98.80%) (Jayant And Vijayakumar 2021).

Bacterial endophytes have also demonstrated promising enzyme-inhibitory potential (Pujiyanto et al. 2018; Pierre et al. 2022). Endophytic bacteria from the roots, stems, and leaves of *Annona muricata* produced α-amylase inhibitors, with isolate DS21 exhibiting the highest inhibition rate of 72.22% (Pujiyanto et al. 2018). Similarly, endophytic bacteria from *Ludwigia octovalvis* showed α-glucosidase-inhibitory activity, with 19 strains isolated from various plant organs, and all extracts displaying measurable activity. One extract, S4155, exhibited greater than 50% inhibition, with an IC_50_ of 163.98 µg/mL, indicating moderate potency relative to established inhibitors. These findings reinforce the importance of bacterial endophytes as reservoirs of bioactive metabolites that can modulate postprandial hyperglycaemia (Pierre et al. 2022).

### Enhancement of GLUT4 translocation

GLUT4 is an insulin-responsive glucose transporter highly expressed in adipose and striated muscle tissues, which are the principal sites of postprandial glucose uptake. Under insulin-stimulated conditions, GLUT4-containing vesicles translocate from intracellular storage compartments to the plasma membrane, where they facilitates glucose diffusion into striated muscle cells and adipocytes (Watson And Pessin 2007; Van Gerwen et al. 2023). This process is primarilyregulated through the PI3K/Akt insulin signalling pathway. Following insulin receptor activation, insulin receptor substrate (IRS) proteins stimulate PI3K activity, leading to phosphatidylinositol-3,4,5-trisphosphate (PIP3) synthesis, and Akt activation, and downstream regulation of GTPase-dependent trafficking events that direct GLUT4 vesicles to the cell surface (Sayem et al. 2018; Camaya et al. 2022).

Endophyte-derived metabolites are increasingly being investigated for their ability to modulate insulin signalling and improve glucose uptake (Agrawal et al. 2022). Bacterial endophytes, including *Bacillus subtilis*, *Streptomyces*, and *Pseudomonas* species, produce peptides, alkaloids, and polyketides that stimulate insulin secretion, modulate GLUT4 translocation, and regulate hepatic gluconeogenesis (Bolivar-Anillo et al. 2021). For example, secondary metabolites from *Pseudomonas protegens* CM-YJ44, an endophytic bacterium isolated from *Dendrobium officinale*, promoted glucose uptake and glycogen synthesis, enhanced pyruvate kinase and hexokinase activities, and restored insulin signalling through the activation of the IRS1/PI3K/AKT/GSK3β/GLUT4 pathway. These metabolites also suppressed pro-inflammatory cytokines and ROS, both of which contribute to insulin resistance (Qin et al. 2025). Molecular docking further identified dendrobine as a putative bioactive component, with strong predicted binding affinity for GLUT4 (binding energy: −7.9 kcal/mol), suggesting potential direct engagement with the glucose transporter (Qin et al. 2025).

Fungal endophytes may also enhance glucose homeostasis through mechanisms that support insulin responsiveness. Metabolites from *Penicillium canescens* and *Pestalotiopsis neglecta*, including xanthones and polyketide-derived compounds, have shown α-glucosidase and protein tyrosine phosphatase 1B (PTP1B)-inhibitory activities. PTP1B is a negative regulator of insulin signalling, therefore, its inhibition may enhance insulin pathway activation and indirectly support GLUT4-mediated glucose uptake (Malik et al. 2020). Endophytic fungi from *Cannabis sativa*, including *Aspergillus micronesiensis* and *Nodulisporium verrucosum*, have also demonstrated inhibitory effects against α-amylase, α-glucosidase, DPP-IV, and lipase, while significantly promoting insulin secretion in pancreatic β-cells (Agrahari et al. 2026). In addition, endophytic metabolites from *Bauhinia variegata* were shown to activate peroxisome proliferator-activated receptors (PPARs) and reduce oxidative stress, mechanisms that may improve insulin sensitivity and glucose homeostasis (Mesquita et al. 2022).

Collectively, the evidence suggests that endophyte-derived metabolites may enhance GLUT4-dependent glucose uptake through both direct and indirect mechanisms. These include activation of IRS1/PI3K/Akt signalling, modulation of GSK3β and GLUT4 expression, inhibition of negative regulators such as PTP1B, stimulation of insulin secretion, and attenuation of oxidative and inflammatory stress that impairs insulin sensitivity. These multifunctional activities support the potential of endophytic microorganisms as sources of antidiabetic compounds targeting insulin resistance and glucose homeostasis (Mesquita et al. 2022).

### Activation of AMPK

AMP-activated protein kinase (AMPK) is a serine/threonine kinase that functions as a key regulator of cellular energy homeostasis (Agius et al. 2020). Once activated, AMPK inhibits ATP consuming anabolic processes including fatty acid and cholesterol biosynthesis, while promoting catabolic pathways that restore cellular energy balance (Hasanvand 2022). In glucose metabolism, AMPK activation enhances peripheral glucose uptake and reduces hepatic glucose production, partly through suppression of gluconeogenic pathways (Chauhan et al. 2022). This mechanism is particularly relevant to diabetes therapy because AMPK activation contributes to the glucose lowering effect of metformin (Agius et al. 2020).

Several natural products exert antidiabetic activity through AMPK-dependent mechanisms, however, evidence for AMPK activation by endophyte-derived metabolites remains comparatively limited. Ranganathan and Mahalingam (2020) investigated 2,4,6-triphenylaniline (TPA), a metabolite derived from *Alternaria longipes* isolated from *Avicennia officinalis* and a TPA-loaded nanoemulsion formulation using in vivo and in silico approaches. Diabetic rats were orally treated with TPA, TPA-loaded nanoemulsion or metformin at 10 mg/kg body weight once daily for 48 days. Both TPA and the TPA-loaded nanoemulsion improved glycaemic control and produced favourable effects on electrolyte balance, liver and kidney function, with responses comparable to metformin. Gene expression analysi indicated that TPA and its nanoemulsion formulation downregulated protein kinase A (PKA) and upregulated AMPK expression, indicating modulation of an energy-sensing pathway s similar that targeted by metformin (Ranganathan And Mahalingam 2020). These findings suggest that AMPK activation may contribute to the antidiabetic activity of selected endophyte-derived metabolites. Nevertheless, additional mechanistic studies are required to confirm AMPK pathway engagement, identify upstream regulatory targets, and determine whether this mechanism is broadly represented among endophytic bioactive compounds.

### Agonism of PPARγ

Peroxisome proliferator-activated receptor gamma (PPARγ) is a ligand-activated nuclear receptor that plays a critical role in adipocyte differentiation, lipid metabolism, insulin sensitivity, and glucose homeostasis. It is also a major pharmacological target of thiazolidinediones, a class of insulin-sensitising antidiabetic drugs that improve glucose tolerance by activating PPARγ-dependent transcriptional pathways (Muralidaran And Roy 2016; Malhotra And Bhatt 2019). Through its regulatory effect on lipid storage in white adipose tissue, adipokine signalling, and peripheral insulin responsiveness, PPARγ contributes to metabolic control in diabetes (Reza-López et al. 2023).

Several endophytic fungi have been reported to produce metabolites with potential PPARγ agonistic activity. El-Sayed et al. (2025) identified multiple forest plant-associated endophytes with putative PPARγ-activating potential, including *Sphaeropsis sapinea* BUK-L2 from *Fagus sylvatica*, *Coniochaeta velutina* SW-B from *Picea abies*, *Epicoccum nigrum* COR-B from *Corylus avellana*, *Paraphaeosphaeria verruculosa* JAR-B from *Sorbus aucuparia*, *Umbelopsis isabelline* COR-L1 from *Corylus avellana*, and *Epicoccum mezzettii* QR-B from *Quercus robur*. These findings suggest that endophytes associated with forest plants may represent underexplored sources of insulin-sensitising metabolites.

Additional evidence was provided by Mesquita et al. (2022) who evaluated metabolites from *Phomopsis* sp. BvFII, an endophyte isolated from *Bauhinia variegata*. in vitro assessmen*t* revealed that this extract activated multiple PRAR isoforms including PPARγ, with activity comparable to the positive control, indicating pan-PRAR agonistic potential (Mesquita et al. 2022). Because PPARγ activation improves insulin sensitivity and contributes to glucose regulation, these findings support the relevance of endophyte-derived metabolites as candidate modulators of nuclear receptor-mediated antidiabetic pathways. However, further compound isolation, receptor-binding studies, dose-response evaluation, and in vivo validation are required to confirm their therapeutic potential.

### Antioxidant protection of pancreatic β-cells

Hyperglycaemia-induced ROS generation contributes to pancreatic β-cell dysfunction, thereby accelerating diabetes progression. Pancreatic β-cells are particularly vulnerable to oxidative injury because they possess relatively low endogenous antioxidant capacity compared with many other cell types. Excessive ROS can impair insulin gene expression, disrupt β-cell function, and promote apoptosis (Gerber And Rutter 2017). Therefore, compounds that reduce oxidative stress, preserve β-cell viability, or enhance β-cell function are of considerable therapeutic interest in diabetes management (Lee et al. 2020).

Endophyte-derived metabolites have shown potential in protecting pancreatic β-cells and supporting insulin secretory function. Ethyl acetate extracts of *Aspergillus micronesiensis* and *Nodulisporium verrucosum*, isolated from *Cannabis sativa*, demonstrated low cytotoxicity, enhanced cell viability, and significantly increased insulin secretion in MIN6 pancreatic β-cells after 48 h of treatment (Agrahari et al. 2026). These findings suggest that metabolites from endophytic fungi may help preserve β-cell function under diabetogenic conditions. Additional evidence comes from xanthone metabolites produced by *Stachybotrys chartarum*, an endophytic fungus isolated from a mangrove plant. These xanthones promoted β-cell mass expansion by stimulating proliferation of existing β-cells and facilitating cell-cycle progression at the G1/S transition (Gan et al. 2020). Because β-cell loss and dysfunction are central features of diabetes progression, strategies that preserve β-cell survival, enhance functional capacity, or stimulate β-cell regeneration may offer benefits beyond symptomatic glucose lowering. Thus, antioxidant and β-cell-protective metabolites from endophytes represent promising leads for antidiabetic drug discovery, although further mechanistic and in vivo validation is needed.

### Anti-inflammatory modulation of TNF-α, IL-6, and IL-1β signalling

Chronic low-grade inflammation contributes to insulin resistance and β-cell dysfunction in diabetes. Pro-inflammatory cytokines, particularly tumour necrosis factor-alpha (TNF-α), interleukin-6 (IL-6), and interleukin-1 beta (IL-1β), interfere with insulin signalling and promote metabolic dysfunction (Berbudi et al. 2025). Therefore, endophyte-derived metabolites that suppress inflammatory mediators may contribute to improved glucose homeostasis through indirect insulin-sensitising and cytoprotective effects (Paramita Pal et al. 2023; Hwang et al. 2025). Several endophyte-derived secondary metabolites have demonstrated anti-inflammatory activity through modulation of cytokine signalling and inhibition of inflammatory enzymes, including inducible nitric oxide synthase (iNOS) and cyclooxygenase-2 (COX-2). A central mechanism involves suppression of the NF-κB pathway, which regulates the transcription of many pro-inflammatory genes. Hwang et al. (2025) reported that pestalotic acid A (PAA), a polyketide derived from the endophytic fungus *Pestalotiopsis vismiae*, suppressed the release of IL-6, IL-1β, and TNF in lipopolysaccharide (LPS)-stimulated RAW264.7 macrophages. Western blot and immunofluorescence analysis revealed that PAA inhibited NF-κB p65 phosphorylation indicating blockade of a key inflammatory signalling node.

Concurrent inactivation of the MAPK and NF-κB pathways has also been reported. Wang et al. (2022) reported that polyketide derivatives from the mangrove endophytic fungus *Daldinia eschscholtzii* KBJYZ-1 effectively suppressed iNOS and COX-2 expression in LPS-stimulated RAW264.7 cells, with further mechanistic analysis revealing that their anti-inflammatory function was exerted by inactivating both the MAPK and NF-κB signalling. Similarly, hypomonacid A, a polyketide from the endophytic fungus *Hypomontagnella* sp. TX-09, isolated from *Santalum album*, inhibited LPS-induced inflammation in mouse macrophage RAW264.7 cells by simultaneously suppressing iNOS, TNF-α, IL-1β, and IL-6 expression without detectable cytotoxicity (Ouyang et al. 2025). At the receptor proximal level, Chen et al. (2021) demonstrated that butyrolactone-I (BTL-1),produced by the coral-associated endophytic fungus *Aspergillus terreus*, significantly inhibited the TLR4/NF-κB signalling pathway and JNK phosphorylation in LPS-induced intestinal epithelial cells, resulting in decreased IL-1β and IL-6 expression. In an in vivo mouse colitis model, the same compound further inhibited MAPK signalling, thereby reducing production of IL-1, IL-6, and TNF-α.

Additional studies support direct modulation of cytokine production by endophytic fungal extracts and purified compounds. Extracts from the endophytic fungus *Alternaria alternata*, isolated from *Calotropis procera* leaves, and *A. terreus*, isolated from *Trigonella foenum-graecum* seeds, counteracted LPS-induced inflammation in THP-1 macrophages, with the *A. alternata* extract reducing TNF-α production by 66% at 20 µg/mL (Spina et al. 2023). Jia et al. (2022) isolated bauvaroalterins A–C from *Alternaria* sp. J030 and demonstrated that these benzylated hydroxyacetophenone derivatives significantly reduced LPS-induced production of TNF-α, IL-1β, and IL-6 production. Their effects were associated with with inhibition of IκB-α phosphorylation and downregulation of NF-κB subunits p50 and p65, indicating suppression of NF-κB activation. Mahana et al. (2023) further demonstrated cytokine-selective effects among metabolites isolated from *A. alternata* endophytes of *Physalis pruinose.* Among these, 3′-hydroxyalternariol monomethyl ether showed strong overall anti-inflammatory activity by suppressing IFN-γ and IL-1β expression, whereas alternariol monomethyl ether was the most potent inhibitor of TNF-α. These differential activities were further supported by molecular docking analyses, which indicated favourable binding affinities between each compound and its respective cytokine target.

Collectively, these findings indicate that endophyte-derived metabolites can attenuate inflammation through multiple molecular targets, including TNF-α, IL-6, IL-1β, iNOS, COX-2, TLR4, NF-κB, JNK, and MAPK signalling. Because inflammation contributes directly to insulin resistance and β-cell stress, these anti-inflammatory effects may complement other antidiabetic mechanisms, including enzyme inhibition, antioxidant protection, and improvement of insulin sensitivity.

### Bioprospecting and discovery strategies

**Fig. 2:**
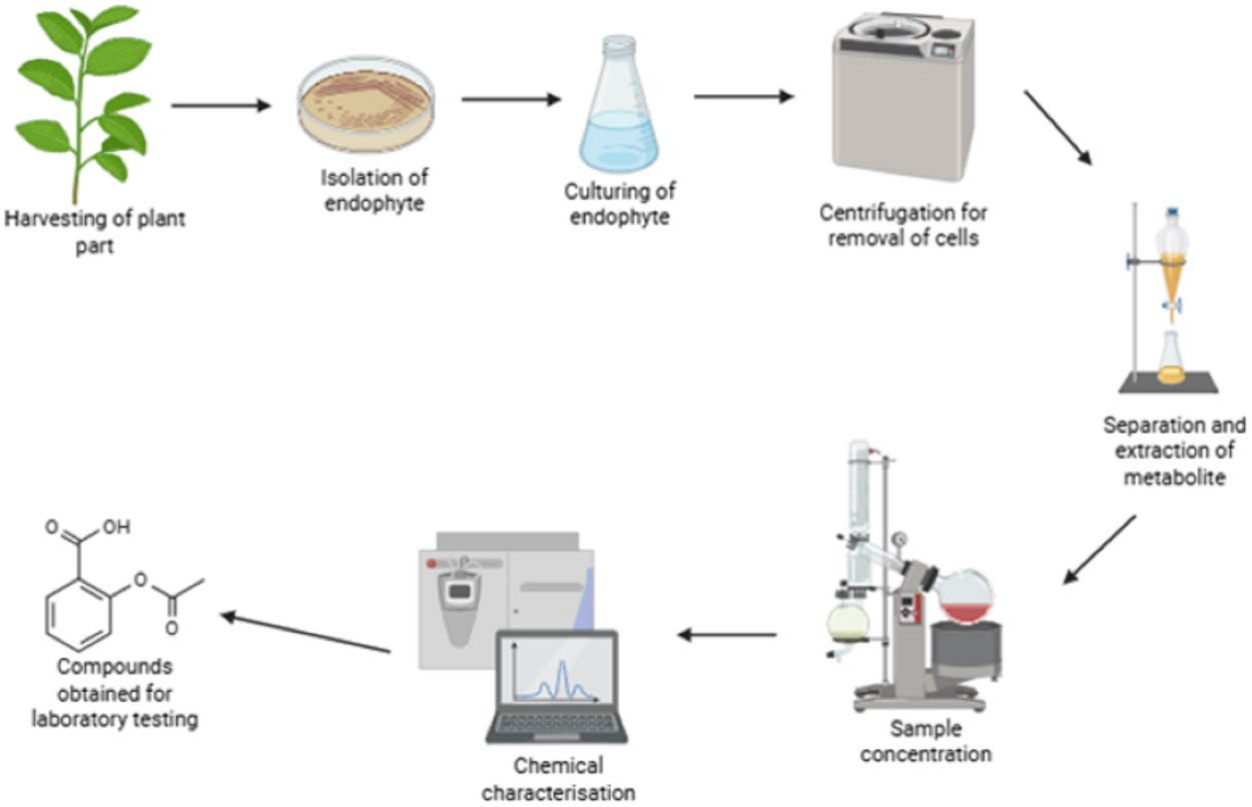
Conventional bioprospecting strategies for endophyte-derived bioactive metabolites. The schematic depicts the workflow involves the isolation of endophytes from surface-sterilised medicinal plant tissues, followed by culturing, biomass production, and solvent-based metabolite extraction. In the illustrated workflow, bioactive secondary metabolites are recovered using a two-step liquid–liquid extraction with diethyl ether. The resulting extracts are then screened for antimicrobial, antidiabetic, anticancer, anti-inflammatory, and antioxidant activities. Figure created using BioRender

**Fig. 3:**
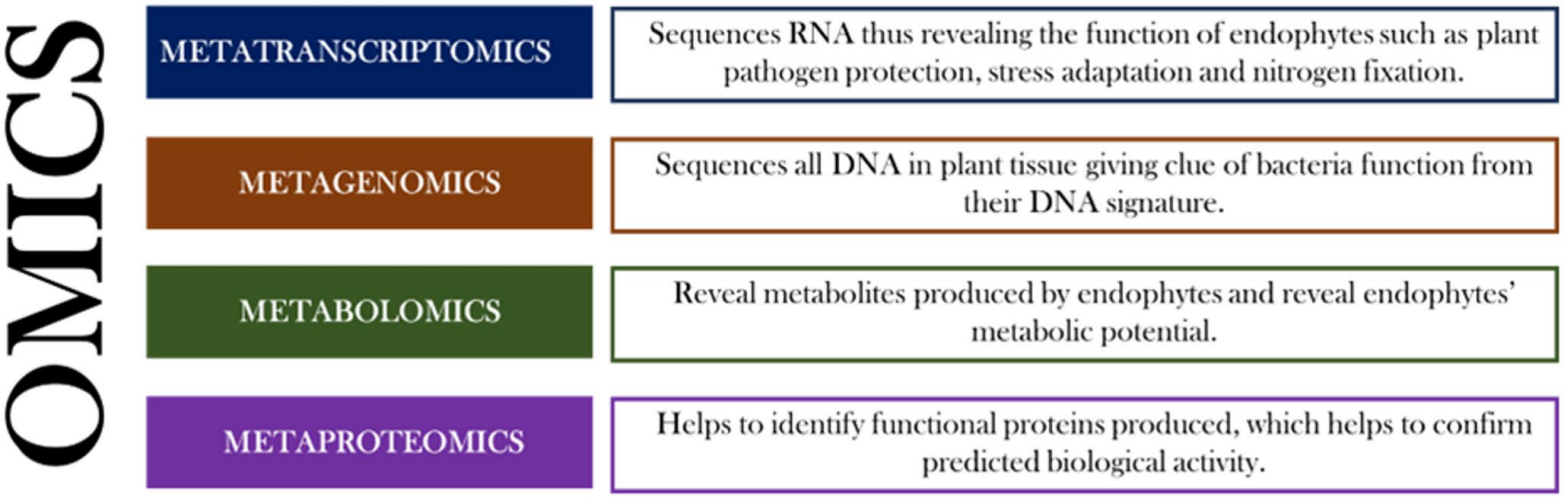
Omics-based identification of non-culturable endophytes. The schematic illustrates how culture-independent omics approaches, including metagenomics, transcriptomics, proteomics, and metabolomics, can be used to detect, characterise, and functionally profile non-culturable endophytes. These methods enable the identification of microbial taxa, biosynthetic gene clusters, expressed pathways, proteins, and bioactive metabolites that may be missed by conventional culture-based techniques

Effective bioprospecting of endophytic microorganisms requires the integration of culture-dependent and culture-independent approaches (Wijayawardene et al. 2021). Conventional methods (Fig. 2) involve the collection of healthy plant tissues, surface sterilisation, selective culturing, purification of fungal or bacterial isolates, and molecular identification (Sahu et al. 2022). However, because only a limited proportion of endophytic diversity is culturable, culture-independent techniques such as metagenomics (Fig. 3) are increasingly used to reveal the taxonomic and functional potential of uncultivable endophytes (Liaqat And Eltem 2016). Functional metagenomics further enables environmental DNA to be expressed in heterologous hosts, thereby facilitating the discovery of novel bioactive compounds (Baliyarsingh 2020). High-throughput screening combined with metabolomics and transcriptomics, has improved the efficiency of evaluating endophytic extracts against disease-relevant targets, including enzymes and signalling pathways implicated in diabetes (Castillo-Olvera et al. 2026). Analytical platforms such as mass spectrometry and nuclear magnetic resonance spectroscopy support metabolite dereplication, structural elucidation and dereplication, and discovery efficiency (Borges And Teixeira 2024). Together, these approaches connect endophytic biodiversity with pharmacological screening and support the identification of candidate metabolites for antidiabetic drug discovery.

The “one-strain many-compounds” (OSMAC) strategy is another important approach for expanding the chemical diversity of endophyte-derived metabolites. It is based on the principle that varying microbial culture conditions, including media composition, aeration, salinity, pH, and enzyme inhibition, can significantly alter secondary metabolite profiles (Hewage et al. 2014). By modifying these parameters, OSMAC can activate otherwise silent or weakly expressed metabolic pathways, increase metabolite yield, and induce the production of novel compounds (Reen et al. 2015; Ahsan et al. 2017).

Current methods for activating silent biosynthetic gene clusters (BGCs) are largely untargeted, meaning they can induce the production of new secondary metabolites without identifying the specific gene cluster responsible (Mao et al. 2018). Genome mining and genetic engineering provide more targeted strategies for accessing cryptic biosynthetic potential. Bioinformatics tools can identify biosynthetic gene clusters (BGCs) in endophytic genomes, after which genetic manipulation may be used to activate, enhance, or heterologously express selected pathways (Covington et al. 2021). Secondary metabolite production is regulated by complex interactions between pathway-specific regulators, located within or near BGCs and global transcriptional regulators that respond to environmental and physiological cues (Macheleidt et al. 2016; Mózsik et al. 2022). Understanding these regulatory networks is essential for improving metabolite reproducibility and scalable production.

Traditional bioprospecting is inherently laborious, involving the collection, isolation, fermentation, extraction, bioassay screening, and structural characterisation of endophytic strains (Fig. 2), a process that historically spans years or decades before yielding clinically actionable leads. Artificial Intelligence (AI) is increasingly accelerating natural product discovery by improving strain prioritisation, scaffold prediction, target identification, and lead optimisation (Mullowney et al. 2023). Machine learning (ML) algorithms, including random forest, support vector machines, deep neural networks, and graph convolutional networks, are being applied in quantitative structure-activity relationship (QSAR) modelling to predict the antidiabetic potential of endophyte-derived compounds prior to synthesis or isolation (Odugbemi et al. 2024). These models are trained on curated datasets of known antidiabetic metabolites and their molecular descriptors, allowing the virtual screening of large chemical libraries derived from endophytic metabolomes. Compounds predicted to exhibit inhibitory activity against validated antidiabetic targets, including α-glucosidase, α-amylase, DPP-IV, PTP1B, and AMPK activators, can be rapidly prioritised for experimental validation, substantially reducing the cost and attrition rate of early-stage drug discovery.

Natural language processing (NLP) tools can further support endophyte bioprospecting by systematically mining large volumes of scientific literature to identify underexplored host plant-endophyte associations, biogeographical patterns, and previously reported bioactivities that may indicate promising avenues for antidiabetic compound discovery (Mullowney et al. 2023). By aggregating and synthesising data across thousands of publications, NLP-powered platforms can identify knowledge gaps, flag high-priority taxa for collection, and generate testable hypotheses that would be impractical to derive solely through manual literature review. Generative artificial intelligence approaches, including variational autoencoders (VAEs) and generative adversarial networks have emerged as powerful tools for *de novo* molecular design by learning latent structural representations of chemical space and generating novel compounds with optimized properties (Kotkondawar et al. 2025; Sun et al. 2025). These models capture the underlying “structural grammar” of known bioactive molecules, enabling the generation of new chemical entities that preserve key pharmacophoric features while simultaneously optimising multiple parameters such as drug-likeness, binding affinity, and synthetic feasibility (Du et al. 2024; Sun et al. 2025). This capability is particularly valuable for natural product–inspired scaffolds, including those derived from endophytes, where generative frameworks can facilitate lead optimisation and exploration of chemical space without reliance on extensive synthetic campaigns, thereby accelerating early-stage drug discovery (Liu et al. 2023; Du et al. 2024).

AI-assisted genomics-guided discovery is also becoming increasingly important. Bioinformatics platforms such as antiSMASH, PRISM, and BiG-SCAPE support the identification, annotation, and clustering of BGCs, predicting the structural class and potential bioactivity of encoded metabolites with increasing accuracy (Hannigan et al. 2019; Walker And Clardy 2021). Furthermore, deep learning models trained on BGC sequence data are beginning to predict the three-dimensional structures of encoded metabolites directly from genomic sequences, bridging the gap between genotype and chemotype with remarkable efficiency (Tryon et al. 2020; Wang et al. 2025). The integration of these genome mining tools with metabolomic databases such as GNPS and the Natural Products Atlas improves dereplication and the targeted prioritisation of potentially novel metabolites (Hannigan et al. 2019; Walker And Clardy 2021).

In diabetes-focused drug discovery, molecular docking and molecular dynamics simulations have been deployed to evaluate interactions between endophyte-derived compounds and antidiabetic targets, such as α-glucosidase, α-amylase, insulin receptor, GLUT4, and PPARγ, (Nagarajan et al. 2025). These computational strategies complement experimental bioassays, providing mechanistic insights into structure-activity relationships and informing the rational optimization of endophyte-derived antidiabetic leads.

### Challenges and limitations in translating endophyte discoveries

Despite growing interest in endophytes as promising sources of bioactive metabolites, their successful translation into clinically relevant therapeutics remains constrained by biological, technical, and regulatory and ethical challenges. A major obstacle is the strain-specific and often unstable nature of secondary metabolite production. Many BGCs remain transcriptionally silent or weakly expressed under conventional in vitro culture conditions, thereby resulting in inconsistent metabolite profiles, poor reproducibility and difficulties in scaling production for pharmaceutical development (Strobel And Daisy 2003). These limitations hinder the establishment of standardised and reliable production platforms, which are crucial for pharmaceutical applications.

The isolation, structural elucidation and characterisation of novel endophyte-derived metabolites also remain technically demanding. Advanced chromatographic, spectroscopic, and metabolomic platforms are required for differentiating novel molecules from known analogues, confirming structural novelty and supporting dereplication (Nafie et al. 2025). However, bioactive metabolites are frequently produced in low quantities, limiting comprehensive chemical characterisation, pharmacological testing, toxicity assessment, and subsequent in vivo validation (Alamgir 2018). Furthermore, many endophytes exhibit slow growth, complex nutritional requirements, or poor adaptation to artificial culture systems, presenting substantial barriers to large-scale fermentation and sustainable metabolite production (Tsipinana et al. 2023).

Beyond technical and biological obstacles, translational progress is also hampered by biosafety, regulatory, intellectual property, and benefit-sharing concerns. Issues related to strain stability, genetic manipulation, environmental release, product standardisation, toxicity profiling, and long-term safety must be addressed before endophyte-derived products can advance toward clinical or commercial application (Nair And Padmavathy 2014). Legal and ethical challenges are also important, particularly where microbial resources are obtained from biodiversity-rich regions. Unresolved questions around ownership, intellectual property rights, access to genetic resources, and equitable benefit-sharing can delay product development and commercialisation (Liu et al. 2024). Addressing these challenges will require standardised cultivation protocols, robust metabolite validation, scalable bioprocessing platforms, clear regulatory pathways, and ethically grounded access and benefit-sharing frameworks.

### Future perspectives and strategies

The future development of endophyte-derived therapeutics will depend on the integration of genomics, biotechnology, synthetic biology and advanced computational approaches. Metagenomic analyses can reveal the genetic potential of uncultivable endophytes and identify cryptic BGCs that may encode novel bioactive metabolites. Complementary transcriptomics and proteomics can clarify regulatory networks, enzymes, and pathway-level mechanisms that control secondary metabolite biosynthesis (Scherlach And Hertweck 2021).

Synthetic biology provides solutions to overcome host dependency and improve metabolite production. The transfer bi of selected BGCs into genetically tractable microbial hosts such as *Escherichia coli* or *Saccharomyces cerevisiae* may enable more stable, scalable, and controllable production of endophyte-derived compounds. In parallel, OSMAC approaches and co-culture systems can simulate environmental stressors and microbial interactions, thereby activating silent pathways and enhancing the diversity of metabolites produced (Pan et al. 2019).

Predictive modelling, AI and genome editing furth are also expected to strengthen endophyte-based drug discovery. Machine learning algorithms can support strain prioritisation, bioactivity prediction, compound dereplication, and identification of metabolites with high therapeutic potential. CRISPR-based genome editing can be used to activate silent BGCs (Ahlawat et al. 2024). Together, these approaches can shorten discovery timelines and increase the reproducibility of candidate lead development.

Successful translation will also require standardised bioassays, robust preclinical validation, scalable fermentation systems, sustainable funding, and interdisciplinary collaboration among microbiologists, natural product chemists, pharmacologists, bioinformaticians, clinicians, and regulatory scientists. Equally important are ethical and legal frameworks that ensure responsible access to microbial resources, equitable benefit-sharing, and protection of biodiversity-rich source regions. With these strategies, endophytes can become a more reliable and sustainable platform for the discovery and development of natural therapeutics.

### Strengths and limitations of the present study

This review possesses several strengths that support its relevance to the field of endophyte-derived antidiabetic therapeutics. First, it provides a structured and comprehensive synthesis of evidence on endophyte-derived antidiabetic metabolites, with emphasis on their biochemical mechanisms, bioprospecting strategies, and translational challenges. The use of a systematic search strategy across four major scientific databases, together with clearly defined search terms, eligibility criteria, and date restrictions, improved literature coverage and methodological transparency. The review also integrates evidence across seven major antidiabetic mechanisms, providing a broad mechanistic perspective on the therapeutic potential of endophytic metabolites. In addition, the inclusion of both fungal and bacterial endophytes from diverse host plants and geographical regions highlights the ecological and biochemical diversity that underpins endophyte-based metabolite discovery. Another strength is the integration of classical pharmacological evidence with emerging discovery technologies, including AI-assisted bioprospecting, CRISPR-mediated activation of biosynthetic gene clusters, genome mining, and multi-omics approaches.

Nevertheless, several limitations should be considered when interpreting the findings. Much of the available evidence remains based on in vitro assays, in silico analyses and in vivo rodent models, with limited human clinical validation of endophyte-derived antidiabetic compounds. Considerable heterogeneity in experimental methodologies, bioassay conditions, IC_50_ reporting, metabolite characterisation, and endophyte identification protocols limits direct comparison across studies and prevents robust quantitative synthesis. Publication bias may also have influenced the evidence base, as studies reporting positive bioactivity are more likely to be published than those reporting weak or negative findings. As this review was designed to map and synthesise existing evidence, it does not provide pooled effect estimates or rank compounds by comparative efficacy. Consequently, conclusions regarding the therapeutic superiority of specific metabolites or endophytic taxa should be interpreted cautiously. Although several promising discovery strategies were identified, the translational pathway from endophyte-derived metabolite discovery to clinically approved antidiabetic therapeutics remains underdeveloped, particularly with respect to standardised validation, large-scale production, regulatory approval, intellectual property management, and clinical testing. Future systematic reviews and meta-analyses focusing on mechanistically comparable datasets will be necessary to generate stronger quantitative evidence and support clinical translation.

## Conclusion

Endophytic microorganisms represent a promising and sustainable source of antidiabetic compounds. Their metabolites can modulate several mechanisms involved in diabetes mellitus, including inhibition of carbohydrate-hydrolysing enzymes, insulin signalling enhancement, oxidative stress mitigation, modulation of inflammatory cytokines and improvement of glucose homeostasis. These multi-target activities highlight the potential of endophyte-derived metabolites as lead compounds for the development of therapeutics that address the complex pathophysiology of diabetes.

Recent advances in genome mining, OSMAC strategies, CRISPR-based pathway activation, multi-omics platforms, synthetic biology and AI-assisted compound prioritisation are accelerating and expanding the discovery and optimisation of novel endophyte-derived metabolites. These technologies can improve strain selection, activate silent biosynthetic pathways, increase metabolite yield, support structural characterisation, and accelerate the identification of compounds with antidiabetic potential.

Despite these advances, several barriers remain before endophyte-derived therapeutics can achieve clinical translation. These include inconsistent metabolite production, strain instability, low compound yield, regulatory complexity, biosafety concerns and unresolved intellectual property and benefit-sharing disputes surrounding microbial resources from biodiversity-rich regions. Overcoming these challenges will require standardised experimental protocols, scalable production platforms, rigorous pharmacological and toxicological evaluation, interdisciplinary collaboration, and equitable policy frameworks.

Overall, endophytic microorganisms offer an ecologically responsible and technologically adaptable platform for antidiabetic drug discovery. With improved validation and translational strategies, they may contribute meaningfully to the development of next-generation therapies for diabetes mellitus. 

## Data Availability

Not applicable.
